# Healthy people with nature in mind

**DOI:** 10.1186/s12889-015-2574-8

**Published:** 2015-12-11

**Authors:** Matilda Annerstedt van den Bosch, Michael H. Depledge

**Affiliations:** Department of Work Science, Business Economics and Environmental Psychology, Swedish University of Agricultural Sciences, Box 88, 23053 Alnarp, Sweden; European Centre for Environment and Human Health, University of Exeter Medical School, Knowledge Spa, Royal Cornwall Hospital, Truro, TR1 3HD UK

**Keywords:** Pro-environmentalism, Automatic mind, Neuro-psychology, Anthropogenic, Behaviour change, Nature-based solutions, Ecosystem degradation, Nudging, Climate change

## Abstract

**Background:**

The global disease burden resulting from climate change is likely to be substantial and will put further strain on public health systems that are already struggling to cope with demand. An up- stream solution, that of preventing climate change and associated adverse health effects, is a promising approach, which would create win-win-situations where both the environment and human health benefit. One such solution would be to apply methods of behaviour change to prompt pro-environmentalism, which in turn benefits health and wellbeing.

**Discussion:**

Based on evidence from the behavioural sciences, we suggest that, like many social behaviours, pro- environmental behaviour can be automatically induced by internal or external stimuli. A potential trigger for such automatic pro-environmental behaviour would be natural environments themselves.

Previous research has demonstrated that natural environments evoke specific psychological and physiological reactions, as demonstrated by self-reports, epidemiological studies, brain imaging techniques, and various biomarkers. This suggests that exposure to natural environments could have automatic behavioural effects, potentially in a pro-environmental direction, mediated by physiological reactions.

Providing access and fostering exposure to natural environments could then serve as a public health tool, together with other measures, by mitigating climate change and achieving sustainable health in sustainable ecosystems. However, before such actions are implemented basic research is required to elucidate the mechanisms involved, and applied investigations are needed to explore real world impacts and effect magnitudes. As environmental research is still not sufficiently integrated within medical or public health studies there is an urgent need to promote interdisciplinary methods and investigations in this critical field.

**Summary:**

Health risks posed by anthropogenic climate change are large, unevenly distributed, and unpredictable. To ameliorate negative impacts, pro-environmental behaviours should be fostered. Potentially this could be achieved automatically through exposure to favourable natural environments, with an opportunity for cost-efficient nature-based solutions that provide benefits for both the environment and public health.

## Background

We are in urgent need of finding methods for protecting public health from the negative effects of climate change. A wide range of approaches will be needed to face the many levels of health effects. Basically, the methods can be of a mitigating character, i.e. acting on factors that cause climate change in the first place such as reducing greenhouse gas (GHG) emissions, or adaptive, i.e. various ways to protect health of already present climate change effects, like establishing early heat wave alarm systems. Both approaches are necessary, but mitigating methods hold a promise to contribute to broader health benefits as it prevents the threat rather than treating the effects once harm is already done. This is at the core of public health work. In addition, this creates co-benefit situations, that is both beneficial to environment and public health. As human behaviour is the cause of climate change a logical approach to mitigation would be to change human behaviour into manners where environmental concerns are included in our actions.

**Fig. 1:**
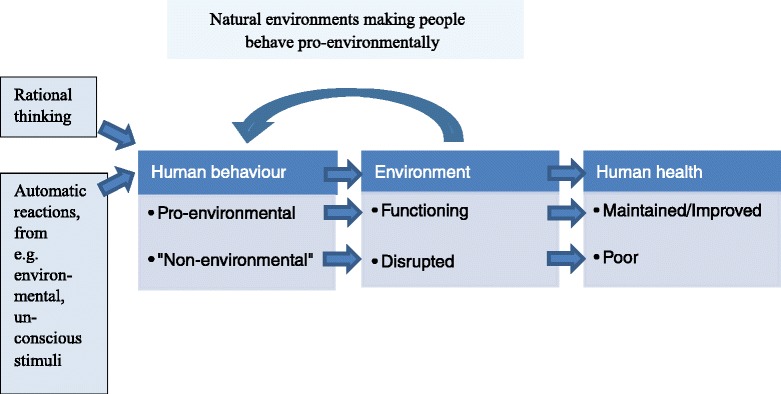
Relation between external stimuli, behaviour, environment and health. Flowchart demonstrating the relationships between factors affecting behaviour, natural environments, and human health, and the direct and indirect consequences of pro-environmental behaviour versus “non-environmental” behaviour for nature and health. The horisontal straight arrows indicate functional associations. The upper arrow, demonstrating a link between natural environments and human behaviour, indicates the hypothesis that exposure to nature has a positive effect on pro-environmental behaviour

This paper will discuss why it is a major task for public health to tackle climate change and how behavioural change may potentially be induced to act as a mitigation tool. We suggest that exposure to nature may induce behavioural change. By drawing on research on humans’ reactions to nature as well as neuroscience for understanding biological fundaments of behaviour, the paper wants to draw attention to this area and how further investigation into the topic could spur research, policies, and actions resulting in improved environmental conditions and public health.

### Healthy planet, healthy people

Climate change is now recognised as one of today’s dominating threats to human health [1]. Alterations in infectious, parasitic and zoonotic diseases, heat- and cold related mortality, and consequences of extreme events provide overt examples of expected health impacts [2, 3]. Insidious, chronic effects of climate change are less well recognised, but may be very significant and will most probably increase over time. Such include for example secondary impacts, like impaired fresh water flow, and tertiary impacts, like health risks associated to escalating resource conflicts [4].

The latest report from the World Health Organization (WHO) concludes that climate change is expected to cause approximately 250 000 additional, unequally distributed deaths annually between 2030 and 2050 [5]. Yet, these figures probably represent underestimations as longer term secondary and tertiary impacts are not accounted for.

### The health sector and the public are waking up at last

Climate change has, by convention, been considered a topic to be dealt with by ecologists and other environmentally related disciplines, and has been underestimated as a serious health issue. The awareness among both health professionals and the public has consequently been insufficient. The direct link to human behaviour has neither been acknowledged. This has resulted in insufficient investments in preparing for climate change impacts on health and an unawareness of the urgent necessity of interactive collaborations between the environmental, behavioural and health sector.

However, as immediate health consequences of climate change are now emerging the topic is gaining increased attention amidst the health sector and in policies [1]. A few studies have also investigated the knowledge of health impact of climate change among the general public, showing that although awareness of health impacts exist the salience is lacking [6]. This demonstrates the value of communicating the health risks that our unsustainable behaviour results in and it has been called for reframing climate change as a public health issue for a better understanding of our dependence on healthy ecosystems [7–9].

### More must be done and novel ideas must be explored

Studies and reports from medicine and public health are increasingly addressing the issue of health impacts from climate change [1, 2, 10–12]. However, much more research is needed, particularly in low-income countries, often the most vulnerable to health effects of climate change [4].

Environmental threats do not fall into individual risk factor categories (in the way that tobacco consumption can be readily studied by, for example, epidemiological methods) but are inherently more complex and elude causality studies and efficient interventions at individual or family levels. In spite of some efforts of interdisciplinary research projects, we do not yet have sufficient knowledge to predict which interventions are most appropriate for particular environmental threats. This obstructs the development of efficient policies and actions for facing and managing current and future environmental challenges in the public health sector.

Many of today’s health care approaches regarding the health impacts of climate change are of a downstream character, based on preparing for what to do when extreme events occur, rather than working upstream to hold disasters at bay. An upstream approach by the health sector to reducing climate change would involve mitigating strategies with possibly much larger and cost-efficient health and environmental benefits.

Engineering and economic system changes for reducing GHG emissions and revert the rapid speed of climate change are promising, but insufficient to meet the climate stabilisation targets of the Sustainable Development Goals (SDGs). Individual and household behaviour changes are also necessary and this is an area for the health sector to urgently start exploring and implying. Dietz et al. [13] showed that environmentally aware household actions could substantially reduce carbon emissions, with little or no impact on general wellbeing.

In a recent editorial on climate change and human survival in the British Medical Journal, McCoy et al. write that health professionals “should each use whatever influence we have to change the minds and behaviour of others” [14]. This is an ethical call for the health sector to adopt a novel model for achieving sustainable health, and resisting and mitigating the severe consequences of climate change. We must learn how to adopt such a model and what ethical tools to use for changing “the minds and behaviour of others”, and not the least ourselves.

### What is the desired behaviour?

Pro-environmental behaviour (PEB) is a behaviour that can have major impact on preserving ecosystems and mitigating climate change [13]. It can be defined as the propensity to take actions and decisions with pro-environmental impact and is commonly understood to be a consequence of attitudes and concerns related to ecosystem destruction, climate change, and other adverse ecological impacts of human activity [15]. PEB is exemplified by behaviours such as decreased use of motorised transport, adopting of recycling, choosing environmentally labelled products, or changing eating habits to more vegetarian food. It can be measured using, for instance, the General Ecological Behaviour (GEB) scale, a reliable and validated measure determining the function of environmental values and intentions, and responsibility feelings [16].

Incorporating efforts towards PEB into public health research and actions, and also explore how such behaviours may be induced, would be one way to reply to the call for health professionals to change human behaviour and actively embrace climate change as part of the medical curriculum.

### How can behaviour be changed?

Behaviour change for the environment has been approached on a societal level through, for example, provision of information, policies, legislations, or enforcements, often with limited success in terms of desired behavioural outcomes and particularly sustained behaviour change is rarely achieved [17]. Although public awareness about climate change is relatively high the behavioural response is small, referred to as the so called “attitude-behaviour” or “value-action” gap [18].

Theories, with more or less empirical support, concerning factors that influence PEB, include the Theory of Planned Behaviour [19], the Value-Belief-Norm [20], and the Theory of Normative Conduct [21]. In research on motivational factors, concepts like “warm glow” or “helper’s high” [22, 23] have been suggested, which link evolutionary adaptive traits of humans (by doing good to others your chance for survival increases and is therefore an inherited quality) to biological development, as the brain reacts on us “doing good” by releasing ‘feel-good’ neurotransmitters, like oxytocin [24]. These concepts have mostly been applied in relation to pro-social behaviour, but links have also been drawn to PEB [25].

### Behaviour change from a biological perspective

Physiological reactions are evoked by internal or external stimuli. These reactions, steered by the brain, result in particular behaviours that are beyond our control of will. For example, stress stimuli automatically induce less cognitively influenced behaviours, by impeding or suppressing activity in the prefrontal cortex (PFC) of the brain [26]. PFC is in charge of higher order cognitive functions, memory, and decision making and stressful stimuli may therefore induce less rationally based behaviours as consequence [27]. Thus, depending on type of stimuli our decision making is unconsciously directed in various ways.

Research in psychology and neuroscience has shown that individual decision-making and subsequent behaviours depend on both rational and non-deliberative, automatic thinking [28, 29]. The rational system is controlled and deductive, while the automatic is uncontrolled, associative and environmentally determined [30]. External stimuli, affecting the automatic system, activates various parts of the brain, determining physiological reactions which in turn guide our behaviour [31].

Most research on human automatic processes has examined social stimuli and social behaviour [25].

Two major neuro-cognitive systems have been proposed as being responsible for socialising behaviour; the mirror neuron system [32] and the mentalizing system [32, 33], especially the mirror neuron system believed to occur automatically [29].

Other studies in the field of social psychology have looked at inducing commitment [16], but to date no consistent solution has been proposed. More importantly, the potential consequences of the theories and changes in behaviour are seldom taken into account in public health policies and activities.

### Environmental stimuli and behaviour change

Compared to social factors or motivators, less research has considered physical environmental stimuli and subsequent automatically induced processes with behavioural impacts. Some studies looking at effects of enriched environments in rat models have revealed that stimulating environments increase levels of neurotrophic factors with consequences for behaviour, rats becoming more socially interactive [34]. This demonstrates how environments with positive cues induce certain behaviours, which might provide a first insight into what environments may be particularly important for automatically changing also human behaviour. However, the underlying mechanisms are unknown and comparable human studies have not been performed.

Other studies on environment and behaviour, defined within the scope of place-based determinants of life-style behaviour, have suggested that physical activity levels can increase by access to healthy environments [35].

Research on concepts like “choice architecture” or “nudging”, have also touched on the idea of how more or less disguised modifications of environmental features influence life-style behaviour [36, 37]. However, far less attention has been devoted to study health effects of behaviours other than those that are directly life-style related.

Recent research, examining behavioural differences along an urban-rural scale has opened a window of understanding on how environments influence neural processing and behaviour. Epidemiological and neuroscientific studies have demonstrated that urban (as compared to rural) dwellers have a higher risk of developing mental disorders [38, 39] and also differ in their capacity to cope with stress as indicated by changes in brain structure and function [40]. There is reason to suspect, therefore, that environmental cues may affect behaviour through automatic reactions initiated by the brain’s responses to the environment, but the area is insufficiently explored and it is unclear which environments might evoke particular behaviours. As urban environments seem to have a specific effect on brain function and behaviour it may be interesting to look into what is known about its counterpart - the natural environment, including urban green spaces.

### Natural environments and automatic behaviour

Natural environments, such as woodlands or urban green spaces, are recognised for their positive effects on stress relief, health, and wellbeing, partly mediated by automatic physiological and neurophysiological reactions [41, 42]. Nature also seems to have an impact on our cognitive function [43]. Even brief encounters with nature and passive exposure may result in immediate effects [44, 45]. A recent brain-imaging study showed that exposure to nature decreases rumination resulting in a reduced risk for depression [46]. The evidence on improved human health and wellbeing by green and natural environments is increasing, showing effects on mental health [47, 48], physical condition with effects on for example cardiovascular health [49], pregnancy outcomes [50], and cognitive and behavioural development [51, 52]. As part of WHO’s environment and health programme, an urban green space indicator has been developed as a proxy for a city’s public health potential [53].

Drawing on these studies, one may suspect that exposure to natural environments can have an immediate, spontaneous behavioural impact through automatic neuro-physiological reaction (see Fig. 1). That this behavioural impact would be in a pro-environmental direction is supported by a recently published study that actually demonstrated that nature exposure may promote environmentally sustainable behaviour, at least in a laboratory setting [54]. However, long-term effects and mechanisms remain to be explored.

Other studies have shown that aesthetically pleasing nature leads to prosocial behaviour [55], which in turn is connected to PEB [56]. Another study revealed that increased concern about marine sustainability was engendered by “nature exposure” through visiting an aquarium [57]. It has also been suggested that feelings of connectedness to, and restoration from nature are linked to PEB [58, 59]. Even childhood experiences in nature appear to enhance adult environmentalism [60] a perturbing fact as children of today spend less and less time outdoors [61].

Much of the research on natural environments’ impact on human beings sets itself within theoretical frameworks referring to humans’ evolutionary development in nature and how this might make us prone to wellbeing and automatically induced stress recovery [62, 63]. Other theories focus on how attention restoration in nature reduces mental fatigue and improves cognitive functioning [64, 65]. Altogether, these theories point in the same direction as suggested above – that nature may indeed induce immediate unconscious and automatic responses. An evolutionary fundament to potential effects on PEB by nature would suggest that the effect could also be retained over time.

## Discussion

### Urbanisation, behaviour change, and future policies

In an increasingly urbanised society contact with nature is diminishing [66]. In urban planning, densification demands threaten parks and other urban green spaces. We know about some of the negative health consequences of this, such as increased prevalence of cardiovascular disease [67] as well as loss of ecosystem services (ESS) for health [68]. Adding the potentially negative effect on PEB by loss of green space makes the health risks even more complex.

Efficient public health policies that manage to change behaviours in an environmentally- friendly direction can make substantial, as well as cost-efficient, long-term contributions to climate-change mitigation and, as a co-benefit, to public health [13].

As results of media campaigns or education programmes are often discouraging we may draw the conclusion that rationality based approaches are probably not the best way forward for changing behaviour that benefits environment and health [69]. Instead we must focus on automatically induced behaviour change and public policies should invest in case studies, programmes and trials on such approaches, in particular for promoting PEB [25]. In this context, we argue that such efforts should stimulate research and actions exploring links between natural environments and PEB, creating an opportunity for nature-based solutions.

### What we need to know

From existing studies we cannot predict what natural environments would induce PEB, to what populations, for how long the effect would remain, or what kind of interactions would be required or even desirable and in what type of settings. The notion of nature as health promoting as such would still encourage studies and attempts to look closer into the matter, especially since interventions would most likely be cost-efficient and with a low risk of harmful effects. There are already policies and programmes at hand which encourage “green planning” for healthier cities [70], though insufficiently implied and the co-benefit of potential PEB and how to optimise this outcome is rarely, if ever, considered.

There is a risk for wear off over time, i.e. if people are continuously exposed to an abundance of nature automatically induced PEB responses would decline It is plausible that by establishing new urban green areas we would get the same effect as by a campaign for PEB, where people would initially respond positively [25], but as the green area becomes the normative environment people would shift to baseline in terms of behaviour. We would argue that this is less likely. Various studies have in general shown sustained effects of for example moving to greener areas [71]. Nature as such is also an inherently changing setting, in terms of for example growth and seasonality and could re-evoke fascination and stimulation over time. The various possible interactions with nature are also close to infinite. However, these matters are essential to investigate before any green investment are promoted, at least if based on a PEB-inducing argument.

Another aspect, similar to studies on health–nature associations, is the self-selection effect. It is possible that people choosing to live in green areas are per se more interested in the environment and thus maintain a higher level of PEB. This calls for carefully designed studies, including both longitudinal investigations as experimental trials.

### All we need is green?

If green spaces are indeed promoting PEB on a general and automatic level, attention should be paid to establish and maintain greenery where it is most needed, such as areas of environmental degradation and where people are less prone to ecological awareness.

From psychology and behavioural research, we know that behaviour is not changed with one intervention [72]. Multiple interventions are required, where nature exposure could only be one. Likewise, we must acknowledge that any measures taken to mitigate climate change and its consequences represent only a small contribution to the wider whole of redirecting our path from a grim future towards one of greater wellbeing in a sustainable and healthy environment. Attempting to increase PEB will not result in a “silver bullet” solution. However, as often is the case in public health interventions, although the individual effect sizes may be small, the consequences for entire populations, and in this case, for the environment too, become substantial.

Even if the evidence on nature’s automatic effect on PEB is lacking, we argue that the topic is worth further exploration for informing future policies on an area calling for innovative solutions – that of climate change and public health.

By implying a “nature-exposure” model for PEB there are also opportunities for “win-win-situations” by adding the ecosystem services provided by green spaces, such as a reduced urban heat island effect [73] and lower air pollution levels [74] to the indirect and long-term positive health effects of PEB. There is even a potential for “win-win-win-situations”, as the use of green spaces in policies and planning offer wider aspects of sustainability, including economic, social, and environmental resilience [75].

## Conclusions

In the health sector there is as yet insufficient recognition that our health is intimately linked to the sustainability of ecosystems, wherein we live our lives. There is also a need for increased understanding of how automatic decisions and behaviour are evoked and the consequences this has for our health and the environment. From the above cited research a few inquiries arise –If the social environment and other external stimuli affect the automatic system of our brains to induce varied kinds of behaviours, is it plausible that stimuli from the physical environment would also generate specific brain reactions automatically spurring us to behave in environmental friendly ways? Can improved accessibility in people’s daily lives to green spaces be used as public health tools by automatically evoking PEB?

This essay is a call for directing resources towards and expanding the public health research agenda to explore environmental factors that automatically may induce PEB and as a consequence improve health. Investigating specifically natural environments, may be a particularly interesting line of research, partly because of existing evidence on nature’s automatic effects on human physiological reactions, but also because of the co-benefits in terms of ecosystem services to be provided by more greenery. Apart from basic research of neuro-anatomical, −functional, and -physiological correlates, there are a wide range of interdisciplinary challenges for applied research to disentangle. We need to know what senses are involved, which environmental factors stimulate the greatest responses, for which behaviours, in which populations, and also how we efficiently and effectively can implement what we learn. These challenges demand our attention now.

## Abbreviations

GEB: General ecological behaviour
GHG: Greenhouse gas emissions
PEB: Pro-environmental behaviour
SDG: Sustainable development goals
WHO: World Health Organization
